# Cordyceps collected from Bhutan, an appropriate alternative of *Cordyceps sinensis*

**DOI:** 10.1038/srep37668

**Published:** 2016-11-22

**Authors:** Ding-Tao Wu, Guang-Ping Lv, Jian Zheng, Qian Li, Shuang-Cheng Ma, Shao-Ping Li, Jing Zhao

**Affiliations:** 1 State Key Laboratory of Quality Research in Chinese Medicine, Institute of Chinese Medical Sciences, University of Macau, Macao, China; 2 National Institutes for Food and Drug Control, Tiantan Xili 2, Beijing, 100050, China

## Abstract

Natural *Cordyceps* collected in Bhutan has been widely used as natural *Cordyceps sinensis*, an official species of Cordyceps used as Chinese medicines, around the world in recent years. However, whether Cordyceps from Bhutan could be really used as natural *C. sinensis* remains unknown. Therefore, DNA sequence, bioactive components including nucleosides and polysaccharides in twelve batches of Cordyceps from Bhutan were firstly investigated, and compared with natural *C. sinensis*. Results showed that the fungus of Cordyceps from Bhutan was *C. sinensis* and the host insect belonged to *Hepialidae* sp. In addition, nucleosides and their bases such as guanine, guanosine, hypoxanthine, uridine, inosine, thymidine, adenine, and adenosine, as well as compositional monosaccharides, partial acid or enzymatic hydrolysates, molecular weights and contents of polysaccharides in Cordyceps from Bhutan were all similar to those of natural *C. sinensis*. All data suggest that Cordyceps from Bhutan is a rational alternative of natural *C. sinensis*, which is beneficial for the improvement of their performance in health and medicinal food areas.

*Cordyceps sinensis*, one of the well-known tonic and traditional Chinese medicines, is a composite consisting of the stromata of the fungus, parasitized on the larva of some species of insects (Family: Hepialidae), and the dead caterpillar^1^,^2^. It is distributed on the Tibetan Plateau and its surrounding regions at an altitude above 3,000 m, including Tibet, Gansu, Qinghai, Sichuan, and Yunnan provinces in China and in certain areas such as the countries of Bhutan, India and Nepal on the southern flank of the Himalayas^1^,^3^,^4^. Usually, *C. sinensis* has been used for the prevention and treatment of a variety of diseases such as asthma, bronchitis, lung inflammation, nocturnal emissions, and night sweats^5^,^6^,^7^. Indeed, nucleosides and polysaccharides were considered as the mainly bioactive components in *C. sinensis*^8^,^9^,^10^,^11^,^12^. Currently, due to its various beneficial effects and limited supply, the price of *C. sinensis* has increased dramatically and is much more expensive, even 4 times, than gold by weight^13^,^14^. Therefore, natural *Cordyceps* collected from Bhutan (Bhutanese Cordyceps) has attracted much attention of the Royal Government of Bhutan, which has been considered as an economically important fungus as natural *C. sinensis*^15^. However, whether Bhutanese Cordyceps could be really used as natural *C. sinensis* remains unknown. Indeed, to the best of our knowledge, few chemical characters of Cordyceps from Bhutan have been investigated, and never been compared with those of natural *C. sinensis*. Therefore, DNA sequence, bioactive components including nucleosides and polysaccharides in twelve batches of Bhutanese Cordyceps were firstly investigated, and compared with natural *C. sinensis*, which are beneficial for better understanding the rational use of Cordyceps from Bhutan.

## Results and Discussion

### Identification of Bhutanese Cordyceps based on DNA barcoding

**Figure 1:**
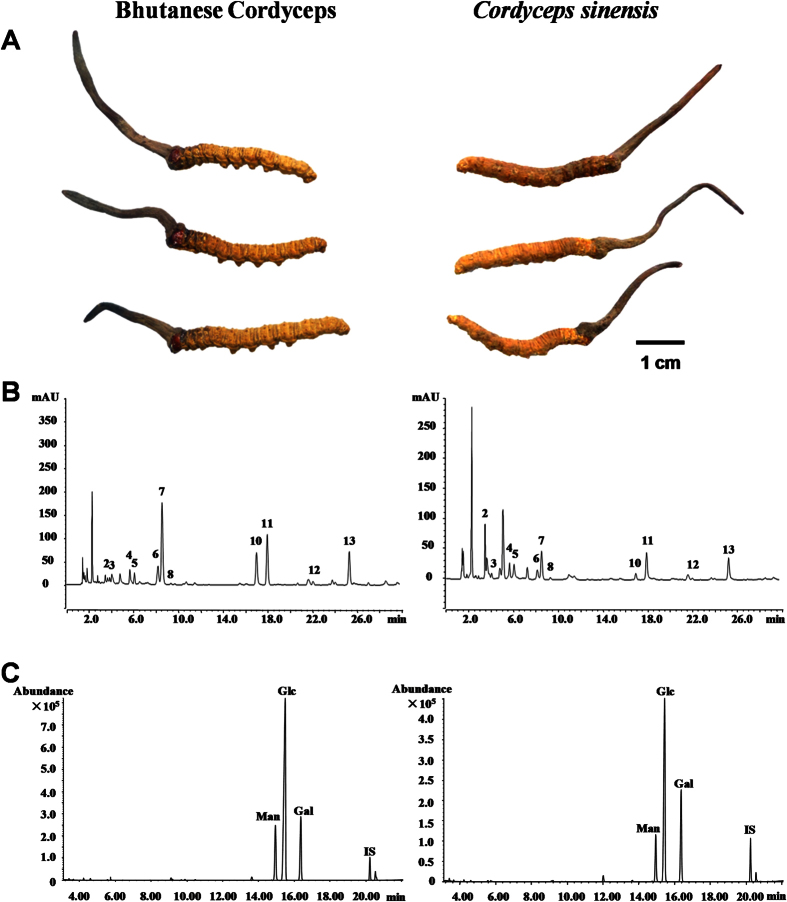
The typical samples (**A**), and representative HPLC-DAD chromatograms of nucleosides (**B**) and GC-MS profiles of compositional monosaccharides of polysaccharides (**C**) from Bhutanese Cordyceps (Left) and natural *C. sinensis* (Right). **2**, uracil; **3**, cytidine; **4**, guanine; **5**, hypoxanthin; **6**, adenine; **7**, uridine; **8**, thymine; **10**, inosine; **11**, guanosine; **12**, thymidine; **13**, adenosine; **Ara**, arabinose; **Fuc**, fucose; **Man**, mannose; **Glc**, Glucose; **Gal**, Galactose; **IS**, internal standard.

Figure 1 showed the typical morphological characteristics of Bhutanese Cordyceps. Compared to natural *C. sinensis*, morphological characteristics of Bhutanese Cordyceps were similar to those of the former^16^, but obvious difference was found in their eyes (Fig. 1A). Therefore, Bhutanese Cordyceps was further identified using DNA barcoding method, which has been widely applied for species identification of animals, plants and fungi^17^,^18^. Indeed, the nuclear ribosomal internal transcribed spacer (ITS) region has been considered as a universal DNA barcode marker for fungi identification^19^. Supplementary Fig. S1A showed the gel electrophoresis of DNA fragments of ITS, Cytb and COI from Bhutanese Cordyceps, and their sequences of stroma and host insect were, respectively, shown in Fig. S1B,C. ITS region of genomic DNA in stroma of Bhutanese Cordyceps was 562 bp, and GC content was 62.13%. According to GenBank NCBI nucleotide database, ITS sequence of Bhutanese Cordyceps was 99% homologous to that of *C. sinensis*. Therefore, the fungus in Bhutanese Cordyceps could be confirmed as *C. sinensis*^1^. In addition, mitochondrial COI and Cytb sequences in host insect of Bhutanese Cordyceps were 658 bp and 433 bp, and GC contents were 29.18% and 22.86%, respectively. Both mitochondrial COI and Cytb sequences suggested that species of host insect of Bhutanese Cordyceps belonged to *Hepialidae* sp (99% homologous)^20^, which was in accordance with those of *C. sinensis*^3^,^21^,^22^.

### Determination of nucleosides in Bhutanese Cordyceps

Although Bhutanese Cordyceps was identified as the same as the species of *C. sinensis*, their chemical characters could be different because of their different locations. Generally, nucleosides and their bases, involved in the regulation and modulation of various physiological processes in body through purinergic and/or pyrimidine receptors^23^,^24^, are considered as the main bioactive components in *C. sinensis*^9^,^25^. To date, more than ten nucleosides and nucleobases, as well as their analogues, including cytosine, uracil, cytidine, guanine, guanosine, hypoxanthine, adenine, adenosine, uridine, thymine, thymidine, 2′-deoxyuridine, inosine and cordycepin have been found in *C. sinensis*^25^. Indeed, adenosine also has been used as a marker for the quality control of *C. sinensis* in Chinese Pharmacopoeia (2015). Therefore, determination of nucleosides and their bases in Bhutanese Cordyceps is extremely important for better understanding its chemical characters and quality.

The typical HPLC-DAD chromatograms of mixed standards and water extract of Bhutanese Cordyceps were shown in Supplementary Fig. S2A and Fig. 1B, respectively. The contents of individual investigated component in Bhutanese Cordyceps were summarized in Table S1. Results showed that types^26^,^27^,^28^ of nucleosides and their bases in water extract, and contents of adenine, uridine, inosine, guanosine and adenosine^27^,^28^,^29^ in Bhutanese Cordyceps were similar to those of *C. sinensis*. In addition, the overall contents of nucleosides are much higher in Bhutanese Cordyceps than those of *C. sinensis* 26,27,29.

### Determination of polysaccharides in Bhutanese Cordyceps

#### Compositional monosaccharides and their molar ratios

Besides nucleosides and their bases, polysaccharides are major contributors to the most of biological activities of *C. sinensis*, and the content of polysaccharides in *C. sinensis* is ranged about 3% to 8% of its total dry weight^8^. Generally, bioactivities of polysaccharides are closely correlated to their chemical structures such as compositional monosaccharides, types of glycosidic linkages, and molecular weight distributions, as well as their absolute content^30^. Therefore, determination of physicochemical properties of polysaccharides in Bhutanese Cordyceps is also extremely important for evaluation of its beneficial effects.

GC-MS analysis has been widely employed for the qualitative and quantitative analysis of compositional monosaccharides in polysaccharides from medicinal plants and fungi^31^. The typical GC-MS profiles of monosaccharides standards and monosaccharides released from polysaccharides in Bhutanese Cordyceps were shown in the Fig. S2B and Fig. 1C, respectively, and their molar ratios were summarized in Table S2. The data showed that compositional monosaccharides of polysaccharides from Bhutanese Cordyceps were mannose, glucose, and galactose, which were in accordance with those of *C. sinensis*^11^,^32^, though their molar ratios in few samples (CC1, CC2, and CC5) of Bhutanese Cordyceps were different to those of *C. sinensis*^32^.

#### PACE and HPTLC fingerprints of partial acid and enzymatic hydrolysates

Saccharide mapping based on HPTLC and PACE analysis has been proven to be a feasible and desirable technique for qualitative analysis of monosaccharides and oligosaccharides released from polysaccharides, which has been successfully applied for partial characterization and comparison of polysaccharides from natural and cultural *C. sinensis* and their related species^33^,^34^. Therefore, partial acid hydrolysates and enzymatic digestions of polysaccharides in Bhutanese Cordyceps were investigated and compared using saccharide mapping based on PACE and HPTLC analysis. Supplementary Fig. S4 showed the HPTLC and PACE fingerprints of hydrolysates of polysaccharides in Bhutanese Cordyceps. Partial acid hydrolysates of polysaccharides from Bhutanese Cordyceps (CC1-CC12), which did not exist in samples before acid hydrolysis (see in the Supplementary Fig. S3), were similar in both PACE and HPTLC fingerprints (Fig. S4A). To improve the specificity, pectinase, α-amylase, and β-D-glucanase were selected for enzymatic digestion of polysaccharides from Bhutanese Cordyceps^34^,^35^. Results showed that both PACE and HPTLC fingerprints of pectinase, α-amylase, and β-D-glucanase digested polysaccharides in Bhutanese Cordyceps were similar (Fig. S4B–D), which suggested that α-1,4-galactosidic, α-1,4-glucosidic, and β-1,4-glucosidic linkages might exist in polysaccharides from Bhutanese Cordyceps^34^,^35^.

#### Molecular weight distributions and contents of polysaccharides and their fractions

HPSEC-MALLS-RID, which has been proven as the powerful and efficient technique for the determination of the molecular weight, molecular weight distribution, as well as contents of polysaccharides and their fractions from natural resources^30^,^36^, was used for the determination of molecular weight distributions and their fractions contents of polysaccharides from Bhutanese Cordyceps. In order to exclude the possible interference from the presence of proteins in the sample solutions, UV absorbance was simultaneously detected at UV 280 nm. The typical HPSEC-RID-UV chromatograms of polysaccharides in Bhutanese Cordyceps were shown in Supplementary Fig. S5, and three peaks were found in samples. Proteins were almost absent in the peaks 1 and peak 2 of all tested samples, while peak 3 had high UV (280 nm) absorbance. Therefore, the molecular weights, polydispersity (*M*_*w*_*/M*_*n*_) and contents of polysaccharide fractions (peak 1 and peak 2) were determined. As shown in Table 1, molecular weights of polysaccharide fractions in Bhutanese Cordyceps were ranging from 1.12 × 10^6^ to 5.51 × 10^6^ Da (peak 1) and 0.45 × 10^5^ to 4.89 × 10^5^ Da (peak 2), respectively. The *M*_w_*/M*_n_ of polysaccharide fraction (peak 1) was ranging from 1.4 to 2.6, while peak 2 was ranging from 1.3 to 1.7. Moreover, total contents of polysaccharides in Bhutanese Cordyceps were ranging from 2.38% to 8.71%, and their average content was 4.53 ± 1.91% (n = 12), which were similar to those of *C. sinensis*^8^.

### Comparison of both nucleosides and polysaccharides in Bhutanese Cordyceps and *C. sinensis*

**Figure 2:**
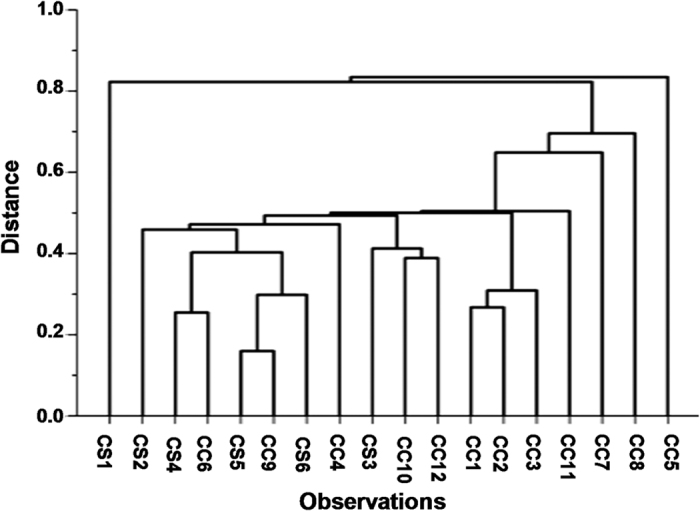
The dendrogram of HCA analysis for all tested samples. HCA analysis of all tested samples was based on their contents of nucleosides and nucleobases, and molar ratios of compositional monosaccharides, molecular weights, and contents of polysaccharides; Sample codes were the same as in Table 1.

Hierarchical clustering analysis (HCA) could calculate the distance matrices of data objects, and organize objects with great similarities into clusters. The applicability of this method has been recognized in many studies^37^,^38^. In order to further investigate the difference and similarity between Bhutanese Cordyceps and *C. sinensis*, HCA was performed based on their nucleosides and polysaccharides analysis, including the contents of the investigated nucleosides and bases, molar ratios of compositional monosaccharides, molecular weights, and contents of polysaccharides. As shown in Fig. 2, almost all samples, except CS1 and CC5, were grouped into one cluster, which suggested that Bhutanese Cordyceps was very similar to *C. sinensis* in active components. However, CS1 and CC5 were grouped into other clusters due to their significant difference in the molar ratio of compositional monosaccharides. The molar ratios of glucose in CS1 and galactose in CC5 were, at least about two folds, much higher than others, respectively, which might attribute to their different locations^32^.

In addition, the professional software named “Similarity Evaluation System for Chromatographic Fingerprint of Traditional Chinese Medicine” (Matlab version, Ver1.315) was used for the evaluation of the similarity of Bhutanese Cordyceps and *C. sinensis* based on their PACE fingerprints of partial acid and enzymatic hydrolysates. The correlation coefficients of individual chromatogram to their simulative mean chromatograms of partial acid, pectinase, α-amylase, and β-D-glucanase hydrolysates of polysaccharides from Bhutanese Cordyceps and *C. sinensis* were summarized in Table 2. The average correlation coefficients of partial acid (SMC-C), pectinase (SMC-P), α-amylase (SMC-A), and β-D-glucanase (SMC-B) hydrolysates of polysaccharides were 0.958 ± 0.021 (n = 18), 0.979 ± 0.011 (n = 18), 0.934 ± 0.024 (n = 18), and 0.965 ± 0.022 (n = 18), respectively. The data further supported that chemical structures of polysaccharides in Bhutanese Cordyceps and *C. sinensis* were similar.

In summary, this study suggests that fungus of Cordyceps from Bhutan is *C. sinensis* and the host insect belongs to *Hepialidae* sp. Their bioactive components, including nucleosides and their bases, and polysaccharides, in Bhutanese Cordyceps are greatly similar to those of *C. sinensis*, which is beneficial for the rational usage of Bhutanese Cordyceps.

## Materials and Methods

### Materials and chemicals

Twelve batches of natural *Cordyceps* were collected from Himalayas of Bhutan (CC1-CC12,), and six batches of natural *C. sinensis* (CS1-CS6) were collected from Tibet and Qinghai of China (Table 1). Identity of natural *C. sinensis* was confirmed by Professor Shao-Ping Li, University of Macau, Macau SAR, China. The voucher specimens were deposited at the Institute of Chinese Medical Sciences, University of Macau, Macao, China.

Cytosine, uracil, cytidine, guanine, hypoxanthine, adenine, uridine, thymine, 2′-deoxyuridine, inosine, guanosine, thymidine, adenosine, and cordycepin were purchased from Sigma (purity ≥ 99.0%, St. Louis, MO, USA). QIAGEN DNeasy plant mini kit, Promega Wizard SV genomic DNA purification system, PrimeSTAR HS DNA Polymerase, and Ex Taq DNA Polymerase were purchased from Takara Biotech Inc. Glucose, mannose, galactose, fucose, arabinose, starch (STN), α-amylase (EC 3.2.1.1), pectinase (EC 3.2.1.15), β-D-glucanase (EC 3.2.1.6), polygalacturonic acid (PGN), dextran (DEN) and acetic anhydride were purchased from Sigma (St. Louis, MO, USA). Laminaribiose (DP2), laminaritriose (DP3), laminaritetraose (DP4), laminaripentaose (DP5), laminarihexaose (DP6) and guar galactomannan (GGN) were purchased from Megazyme (Wicklow, Ireland). Polyacrylamide containing a ratio of acrylamide/N,N-methylenebisacrylamide (19:1, w/w) was obtained from Bio-Rad (Hercules, CA, USA). Silica gel 60 TLC plates were obtained from Merck (Merck, Darmstadt, Germany). Deionized water was prepared by a Millipore Milli-Q Plus system (Millipore, Bedford, MA, USA). All the other reagents were of analytical grade.

### DNA barcoding analysis

DNA extraction, amplification and sequencing were performed according to our previously reported methods with modification^39^,^40^. In brief, specimens of Bhutanese Cordyceps were divided into stroma and host insect. Genomic DNA in stroma and host insect was then isolated using a QIAGEN DNeasy plant mini kit and a Promega Wizard SV genomic DNA purification system, respectively. The ITS regions of genomic DNA in stroma were amplified with a forward primer of ITS5 (5′-GGAAGTAAAAGTCGTAACAAGG-3′) and a reverse primer of ITS4 (5′-TCCTCCGCTTATTGATATGC-3′). The samples were amplified using an ABI Veriti PCR (Applied Biosystems, USA) under the following conditions, initial denaturation at 95 °C for 4 min, followed by 30 cycles of denaturation at 95 °C for 0.5 min, annealing at 52 °C for 0.5 min, extension at 72 °C for 1 min, and a final elongation step at 72 °C for 7 min. In addition, both COI and Cytb sequences of genomic DNA in host insect were also amplified. The COI sequence was amplified with two primers including COI-F (5′-GGTCAACAAATCATAAAGATATTG-3′) and COI-R (5′-TAAACTTCAGGGTGACCAAAAAAT-3′), and the samples were amplified under the following conditions, initial denaturation at 95 °C for 4 min, followed by 35 cycles of denaturation at 95 °C for 0.5 min, annealing at 50 °C for 0.5 min, extension at 72 °C for 1 min, and a final elongation step at 72 °C for 7 min. Moreover, the Cytb sequence was amplified with two primers including Cytb1 (5′-TATGTACTACCATGAGGACAAATATC-3′) and Cytb2 (5′-ATTACACCTCCTAATTTATTAGGAAT-3′), and the samples were amplified under the following conditions: initial denaturation at 95 °C for 4 min, followed by 40 cycles of denaturation at 95 °C for 0.5 min, annealing at 48 °C for 1.0 min, extension at 72 °C for 1 min, and a final elongation step at 72 °C for 7 min. After all PCR products were confirmed by 1.5% agarose gel electrophoresis, the fragments were purified, and then sequenced with the help of INVITROGEN TRADING (SHANGHAI) CO., LTD. Finally, the sequences of ITS, COI and Cytb were blasted against the GenBank NCBI nucleotide database online (http://blast.ncbi.nlm.nih.gov/Blast.cgi?PROGRAM=blastn&PAGE_TYPE=BlastSearch&LINK_LOC=blasthome), respectively.

### Sample preparation

Samples were carefully cleaned without water by a small brush, dried at 40 °C for 24 h, and pulverized via grinding. Nucleosides and their bases in sample materials were extracted using boiling water extraction according to our previously reported method with minor modification^28^. In brief, the powder of Cordyceps (0.5 g) was mixed with 20 mL Milli-Q water in a glass tube, accurately weighted, and placed onto a Syncore Reactor (BUCHI-Syncore, Flawil, Switzerland) and heat reflux (100 °C) for 60 min. After extraction, the extract was cooled down to the room temperature, and made up the lost weight with water, then centrifuged (4000 × g for 5 min). The supernatant was filtered through a 0.45 μm Econofilter before HPLC-DAD analysis.

Water soluble polysaccharides were extracted with microwave assisted extraction according to a previously reported method with minor modification^41^. In brief, the powder of sample materials was immersed in water (20 mL), and extracted with microwave assisted extraction (Multiwave 3000, Anton paar GmbH, Graz, Austria). The microwave irradiation program was performed at 600 W and 90 °C for 20.0 min. Then the extract solution was centrifuged at 4000 × g for 10 min (Allegre X-15 centrifuge; Beckman Coulter, Fullerton, CA, USA). Subsequently, ethanol was added to the final concentration of 80% (v/v) for precipitation of crude polysaccharides. After standing for 12 h at 4 °C, centrifugation (4000 × g for 10 min) was performed. The precipitate was redissolved in 10 mL of hot water (60 °C). After centrifugation (4000 × g for 15 min), the supernatant was collected and the powder of the supernatant was obtained by freeze-drying.

### HPLC-DAD analysis

Qualitative and quantitative analysis of nucleosides and their bases was performed on an Agilent Series 1200 (Agilent Technologies, USA) liquid chromatography system according to a previously reported method with minor modification^29^. In brief, a grace prevail select C18 column (4.6 × 150 mm, 3 μm) was used. The column temperature was maintained at 25 °C. The standards and samples were separated using a gradient mobile phase consisting of water (A) and acetonitrile (B). The gradient condition is: 0–6 min, 0% acetonitrile; 6–20 min: 0–5% acetonitrile; 20–30 min 5–25% acetonitrile. The flow rate was 1.0 mL/min and the injection volume was 10.0 μL. Peaks were detected at 260 nm of UV detection.

### GC-MS analysis

Compositional monosaccharides of polysaccharides were investigated using GC-MS analysis according to a previously reported method with minor modification^11^. Briefly, the sample (~4.0 mg/mL, 0.5 mL) was hydrolyzed with 2.0 M trifluoroacetic acid under microwave irradiation (Multiwave 3000, Anton paar GmbH, Graz, Austria). The microwave irradiation program was performed at 300 W for 6 min. After hydrolysis, the hydrolysates were evaporated to dryness by using nitrogen and washed with methanol for three times to remove the residue of trifluoroacetic acid. Subsequently, 0.5 mL of pyridine and 10.0 mg of hydroxylamine hydrochloride were added and incubated at 90 °C for 30 min, 0.5 mL of acetic anhydride was then added and incubated at 90 °C for 30 min. Furthermore, the derivatives of mixed monosaccharide standards (Ara, Fuc, Gal, Glc, and Man, respectively) were prepared as described above. The derivatives were analyzed by using an Agilent 6890 gas chromatography instrument coupled to an Agilent 5973 mass spectrometer (Agilent Technologies, Palo Alto, CA) according to our previously reported method^11^. In brief, a capillary column (30 m × 0.25 mm, i.d.) coated with 0.25 μm film 5% phenyl methyl siloxane was used for separation. The column temperature was set at 165 °C and held for 7 min for injection, then programmed at 5 °C/min to 185 °C and held for 5 min, then at 4 °C/min to 200 °C, and finally at 20 °C/min to 280 °C, and held for 2 min.

### Saccharide mapping analysis

#### Partial acid and enzymatic hydrolysis of refined polysaccharides

The crude polysaccharides of each sample (40.0 mg) were redissolved in 10.0 mL of hot water (60 °C). Then the low molecular weight compounds were removed by centrifugation (3500 × g, 25 min) with an ultra centrifugal filter (molecular weight cutoff: 3 kDa, Millipore, Billerica, MA, USA) for seven times. Finally, the concentration of crude polysaccharides in each sample was adjusted to the same concentration for further partial acid and enzymatic hydrolysis.

Polysaccharide solutions (~2.0 mg/500 μL) were treated with trifluoroacetic acid at a final concentration of 0.5 mol/L in a total volume of 1000 μL, and incubated at 80 °C for 5 h according to our previously reported method with minor modification^34^. After hydrolysis, the hydrolysates were washed with methanol and evaporated to dryness with a nitrogen evaporator at 35 °C for three times to remove the residue trifluoroacetic acid. The dried products were stored in 4 °C before derivatization, and redissolved in 100 μL of ethanol (70%, *v/v*) for HPTLC analysis, respectively.

In addition, polysaccharide solutions (~2.0 mg/ 500 μL) were mixed with selected enzyme (the final concentration of β-D-glucanase, α-amylase and pectinase was 2, 20 and 20 U/mL respectively) in a total volume of 1000 μL and digested overnight (16 h) at 40 °C. Then the mixtures were heated at 80 °C for 30 min to denature the enzymes. The supernatants were evaporated to dryness with a nitrogen evaporator and then were used for derivatization, and redissolved in 100 μL of ethanol (70%, *v/v*) for HPTLC analysis, respectively. Polysaccharide solution without enzymes, treated as described above, was used as blank control. Subsequently, the partial acid and enzymatic digestions were derivatized with ANTS at 37 °C for 17 h according to a previously reported method^41^.

#### Saccharide mapping based on PACE analysis

All samples (1–8 μL depending of the sugar concentration) were separated using a vertical slab gel electrophoresis apparatus, Mini-Protean Tetra System (Bio-Rad, Hercules, CA, USA) according to a previously reported method^34^. In brief, electrophoresis of 30% (w/v) polyacrylamide in the resolving gel with a stacking gel of 8% (w/v) polyacrylamide was used for the separation of partial acid and enzymatic hydrolysates, respectively. The samples were electrophoresed first at 200 V for 15 min and then at 700 V for 45 min, to move bromophenol blue (migration indicator) to the desired level. Gels were imaged using an InGenius LHR CCD camera system (Syngene, Cambridge, UK) under UV 365 nm.

#### Saccharide mapping based on HPTLC analysis

All the samples (4–10 μL depending of the sugar concentration) were separated on a silica gel 60 plate with an AS30 HPTLC Applicator (Desaga GmbH, Germany) according to a previously reported method^34^. In brief, the bands were 8 mm wide, 13 mm distance, and 10 mm from the bottom edge. Then the plate was firstly developed to a distance of 95 mm with 1-butanol/isopropanol/acetic acid/water, 7:5:1:2 (v/v/v/v) as mobile phase at room temperature. Then the plate was dried and placed in the same chamber to develop a distance of 95 mm with the same mobile phase as described above. Finally, the developed plates were dried and colorized with aniline-diphenylamine-phosphoric acid solution, then heated at 105 °C for 10 min on a YOKO-XR plate heater (Wuhan YOKO technology Ltd., China) and photographed under white night.

### HPSEC-MALLS-RID analysis

The molecular weights (*M*_w_), molecular weight distributions and contents of polysaccharides and their fractions in Bhutanese Cordyceps and *C. sinensis* were measured using HPSEC-MALLS-RID according to a previously reported method with minor modification^36^. In brief, HPSEC-MALLS-RID measurements were carried out on a multi-angle laser light scattering (DAWN HELEOS, Wyatt Technology Co., Santa Barbara, CA, USA) with an with an Agilent 1100 series LC/DAD system (Agilent Technologies, Palo Alto, CA, USA) equipped with a column of TSK-Gel G5000_PWXL_ (300 mm × 7.8 mm, i.d.) and TSK-Gel G3000pw_XL_ (300 mm × 7.8 mm, i.d., Tosoh Bioscience, Tokyo, Japan) in series at 35 °C. A refractive index detector (RID, Optilab rEX refractometer, DAWN EOS, Wyatt Technology Co., Santa Barbara, CA, USA) was simultaneously connected. The *M*_w_ was calculated by the Zimm method of static light scattering based on the basic light scattering equation according to our previously reported method^11^. The content of polysaccharides was calculated based on the refractive index difference with *dn/dc* value according to the following equation^42^,


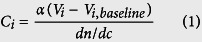


where *C*_*i*_ is the concentration of polymers; *α* is the RID calibration constant (in RI units per volt), which is determined as 3.4756 × 10^−5^ RIU/pixel using the aqueous solutions of reference standard (sodium chloride); *V*_*i*_ and *V*_*i, baseline*_ are the RID voltages of sample and baseline, respectively; *dn/dc* is the specific refractive index increment of polysaccharides, which is defined as 0.15 mL/g according to our previously reported method^36^.

The mobile phase was 0.9% NaCl aqueous solution at a flow rate of 0.5 mL/min. All of polysaccharide solutions were filtered by a 0.22 μm membrane before use. The injection volume was 50 μL for each sample. The Astra software (Version 6.0.2, Wyatt Tech. Corp. Santa Barbara, CA, USA) was utilized for data acquisition and analysis.

### Data analysis

Hierarchical cluster analysis (HCA) was performed by using Origin86 software, the nearest neighbor cluster method with euclidean distance type was selected as measurement for hierarchical clustering analysis. In addition, the optical densities of bands in electronic images and digital scanning profiles of PACE analysis were generated and analyzed using Quantity-One software (version 4.6.2, Bio-Rad, Hercules, USA). The similarities of the tested samples, as well as the simulative mean chromatogram were calculated and generated using the professional software named “Similarity Evaluation System for Chromatographic Fingerprint of Traditional Chinese Medicine” (Matlab version, Ver1.315, developed by the Research Center of Modernization of Chinese Herbal Medicine, Central South University, and the Hong Kong Polytechnic University).

## Additional Information

**How to cite this article**: Wu, D.-T. *et al*. Cordyceps collected from Bhutan, an appropriate alternative of *Cordyceps sinensis. Sci. Rep.* **6**, 37668; doi: 10.1038/srep37668 (2016).

**Publisher’s note:** Springer Nature remains neutral with regard to jurisdictional claims in published maps and institutional affiliations.

## Supplementary Material

Supporting Information

## Figures and Tables

**Table 1:** Molecular weights and contents of polysaccharides in raw materials of Bhutanese Cordyceps and natural *Cordyceps sinensis*.

Sample Codes	Origins	Peak 1^a^			Peak 2^a^			Total Content (%)
		*M*_w_ × 10^6^ (Da)	*M*_w_*/M*_n_	Content (%)	*M*_w_ × 10^5^ (Da)	*M*_w_*/M*_n_	Content (%)	
CS1	China	4.12	2.3	4.85	3.58	1.3	2.99	7.84
CS2	China	1.36	1.8	1.40	1.15	1.4	2.10	3.50
CS3	China	4.75	1.6	2.15	3.79	1.4	1.99	4.14
CS4	China	2.18	1.7	2.55	1.48	1.5	2.38	4.93
CS5	China	3.26	1.8	1.45	1.41	1.4	2.38	3.82
CS6	China	3.28	1.7	2.41	2.09	1.4	2.23	4.64
CC1	Bhutan	1.45	2.2	0.24	0.47	1.4	2.14	2.38
CC2	Bhutan	1.12	1.6	0.35	0.45	1.4	2.94	3.30
CC3	Bhutan	1.36	1.5	0.85	0.54	1.5	3.60	4.45
CC4	Bhutan	4.56	1.6	0.61	1.38	1.5	2.60	3.20
CC5	Bhutan	1.42	2.6	0.23	0.49	1.4	2.24	2.47
CC6	Bhutan	2.85	1.9	2.42	1.45	1.4	2.84	5.26
CC7	Bhutan	1.13	1.8	2.51	0.51	1.7	6.20	8.71
CC8	Bhutan	3.81	1.6	3.24	4.89	1.3	3.46	6.70
CC9	Bhutan	3.02	1.8	1.56	1.48	1.4	2.66	4.21
CC10	Bhutan	4.33	1.4	1.82	3.20	1.4	2.36	4.18
CC11	Bhutan	1.80	2.3	2.06	0.76	1.6	4.24	6.30
CC12	Bhutan	5.51	1.6	0.98	2.81	1.4	2.17	3.15

^a^All data were the average of two measurements with coefficient of variation <4%.

**Table 2:** The correlation coefficient of each tested sample to their simulative mean chromatogram (SMC).

Samples	Simulative mean chromatograms of partial acid and enzymatic hydrolysates			
	Partial acid	Pectinase	α-amylase	β-glucanase
	SMC-C	SMC-P	SMC-A	SMC-B
CS1	0.941	0.970	0.982	0.974
CS2	0.974	0.979	0.926	0.978
CS3	0.947	0.960	0.902	0.947
CS4	0.971	0.990	0.916	0.927
CS5	0.974	0.989	0.907	0.937
CS6	0.983	0.982	0.913	0.975
CC1	0.919	0.968	0.899	0.962
CC2	0.919	0.977	0.901	0.967
CC3	0.956	0.991	0.924	0.910
CC4	0.981	0.996	0.948	0.983
CC5	0.925	0.996	0.954	0.982
CC6	0.986	0.968	0.931	0.970
CC7	0.971	0.980	0.951	0.973
CC8	0.971	0.982	0.949	0.970
CC9	0.971	0.976	0.954	0.967
CC10	0.952	0.958	0.952	0.965
CC11	0.959	0.986	0.964	0.988
CC12	0.946	0.990	0.955	0.992
Average ± SD	0.958 ± 0.021	0.979 ± 0.011	0.934 ± 0.024	0.965 ± 0.022

**SMC-C**, **SMC-P**, **SMC-A** and **SMC-B**, simulative mean chromatograms of partial acid hydrolysates, and pectinase, α-amylase, and β-D-glucanase digested polysaccharides, respectively. The sample codes were the same as in Table 1.
