# Supplementary material for: Cordyceps collected from Bhutan, an appropriate alternative of Cordyceps sinensis
Source: Sci Rep. 2016 Nov 22;6:37668. doi: 10.1038/srep37668 (PMC5118747; doi:10.1038/srep37668)
Supplement: Supporting Information [file srep37668-s1.pdf]

Supporting Information

Cordyceps collected from Bhutan, an approprate alternative of *Cordyceps sinensis*

Ding-Tao Wu [1], Guang-Ping Lv [1], Jian Zheng [2], Qian Li [2], Jing Zhao [1,*], Shuang-Cheng Ma [1,2,*], Shao-Ping Li [1,*]

[1] State Key Laboratory of Quality Research in Chinese Medicine, Institute of Chinese Medical Sciences, University of Macau, Macao, China

[2] National Institutes for Food and Drug Control, Tiantan Xili 2, Beijing 100050, China

* Corresponding authors: Tel: +853 8822 4692; Fax: +853 2884 1358.

E-mail address: jingzhao@umac.mo (J. Zhao), masc@nifdc.org.cn (S.C. Ma), spli@umac.mo or lishaoping@hotmail.com (S.P. Li).

A

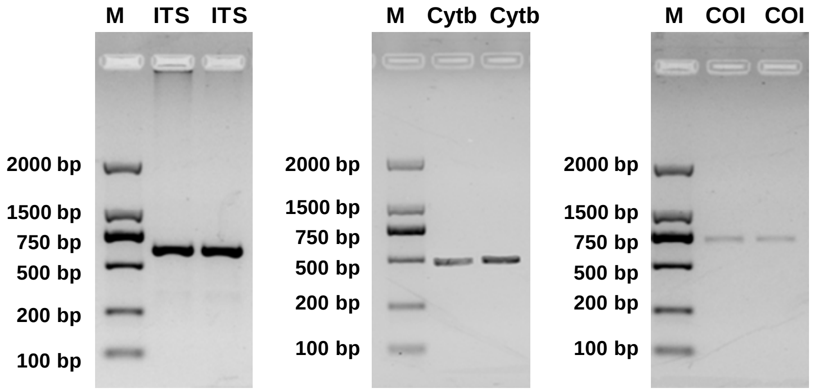

B

# ITS sequence

```
TCTCCGTTGG TGAACCAGCG GAGGGATCAT TATCGAGTTG CCACTCCCAA ACCCCCTGCG   60
AACACCACAG CAGTTGCCTC GGCGGGACCG CCCCGGCGCC CCAGGGCCCG GACAAGGGCG  120
CCCGCCGGAG GACCCCCAGA CCCCCCTGTC GCAGTGGCAT CTCTCAGTCA AGAAGCAAGC  180
AAATGAATCA AAACTTTCAA CAACGGATCT CTTGGTTCTG GCATCGATGA AGAACGCAGC  240
GAAATGCGAT AAGTAATGTG AATTGCAGAA TTCAGTGAAC CATCGAATCT TTGAACGCAC  300
ATTGCGCCCG CCAGCACTCT GGCGGGCATG CCTGTCCGAG CGTCATCTCA ACCCTCGAGC  360
CCCCCGCCTC GCGGCGGCGG GGCCCGGCCT TGGGGGTCAC GGCCCAGCGC CGCCCCCTAA  420
ACGCAGTGGC GACCCCGCCG CGGCTCCCCT GCGCAGTAGC TCACTGAGAA CCTCGCACCG  480
GAAGCGCGGA GGCGGTCACG CCGTGAAACC ACCACGCCCT CCAGTTGACC TCGGATCAGG  540
TAGGGATACC CGCTGAACTT AA                                           562
```

C

# Cytb sequence

```
ATTTTGAGGA GCTACTGTTA TTACAAATTT ATTATCAGCA TTACCTTACT TAGGATCAAT   60
ATTAGTTAAT TGAATTTGAG GTGGTTTTGC AGTAGATAAT GCTACATTAA CTCGATTTTA  120
TACTTTTCAT TTTTTATTAC CCTTTATTTT AATAATAATA ACTATAATTC ATTTATTATT  180
TCTACATCAA TACGGTTCAA ATAACCCATT AGGAATTAAT AGAAATTTAG ACAAAATTCC  240
ATTTCATCCT TATTTTTCAT TTAAAGATTT ATTTGGATTT ATTATTATAA TAATAATTTT  300
AATATTATTA ACATTATGAA ATCCATATAT TTTAAGAGAC CCTGACAATT TTATTCCAGC  360
AAATCCTTTA ATAACTCCAA TTCATATTCA ACCAGAATGA TATTTTTTAT TTGCTTACGC  420
AATTTTACGA TCA                                                     433
```

# COI sequence

```
AAATAAATGT TGGTATAGAA TAGGGTCACC ACCCCCTGCA GGATCAAAAA ATGAAGTATT   60
TAAATTTCGA TCTGTTAATA GTATAGTAAT AGCTCCTGCT AATACAGGTA ATGATAATAA  120
TAGTAATAAT GCTGTAATTA CAACTCTTCA TACAAATAAA GGTATACGAT CTAAAGATAT  180
TCTTTTTGAT CGTATATTAA TTACAGTAGT AATAAAATTA ATTGCTCCTA AAATTGATGA  240
AATTCCTGCT AAATGTAATG AAAAAATAGC TAGATCTACA GATGCTCCAG AATGTGAAAT  300
ATTTGAGGAT AATGGAGGAT AAACAGTTCA ACCTGTTCCT GCTCCGTTTT CTACAATTCT  360
TCTAGAAATT AATAATATTA ATGATGGTGG TAATAATCAA AATCTTATAT TATTAAGTCG  420
AGGGAATGCT ATATCGGGGG CTCCTAATAT TAAAGGAATT AGTCAATTTC CAAAACCACC  480
AATTATAATT GGCATTACCA TAAAAAAAAT TATAATGAAA GCGTGAGCTG TGACAATTAC  540
ATTATAAATT TGATCATCTC CAATTAAAGA TCCGGGGTTT CCTAATTCTA TTCGGATTAA  600
TATTCTTAAA GATGTTCCTA CTATTCCTGC TCAAATACCA AAAATAAAAT ATAAAGTT    658
```

Fig. S1. Agarose gel electrophoresis (A) of ITS, Cytb and COI fragments, and the sequences of ITS (B), Cytb and COI (C) in genomic DNA, respectively, from the stroma and host insect of Bhutanese Cordyceps

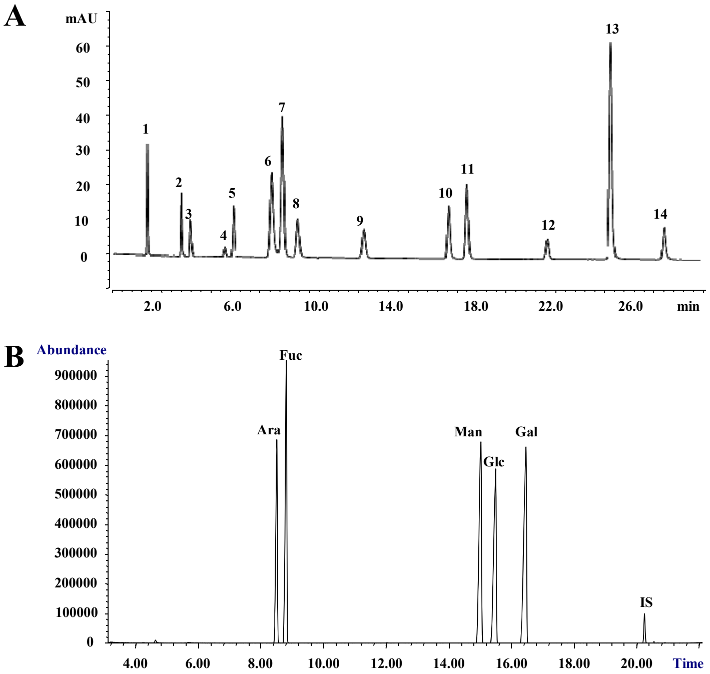

Fig. S2. HPLC-DAD chromatograms of mixture standards of nucleosides (A) and GC-MS profiles of mixture standards of monosaccharides (B)

1, cytosine; **2**, uracil; **3**, cytidine; **4**, guanine; **5**, hypoxanthin; **6**, adenine; **7**, uridine; **8**, thymine; **9**, 2′-deoxyuridine; **10**, inosine; **11**, guanosine; **12**, thymidine; **13**, adenosine; **1**4, cordycepin

Ara, arabinose; **Fuc**, fucose; **Man**, mannose; **Glc**, Glucose; **Gal**, Galactose; **IS**, internal standard;

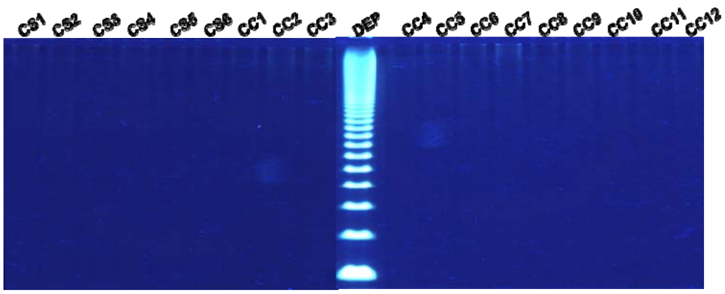

Fig. S3. PACE fingerprints of polysaccharides from Bhutanese Cordyceps and natural *C. sinensis*

DEP, the partial acid hydrolysates of dextran used as marker;

The sample codes were the same as in Table S1.

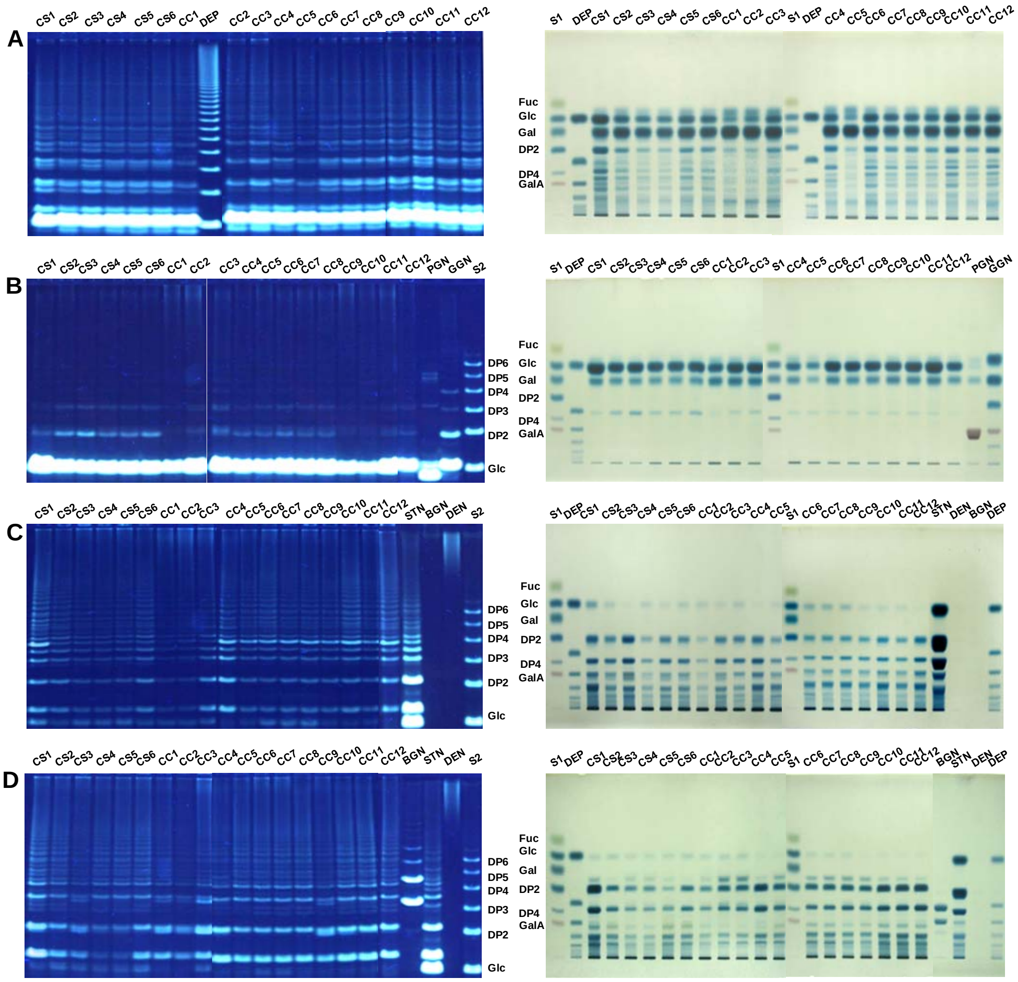

Fig. S4. PACE fingerprints (left) and HPTLC profiles (right) of partial acid (A), pectinase (B), α-amylase (C) and β-glucanase (D) hydrolysates of polysaccharides from Bhutanese Cordyceps and natural *C. sinensis*

S1, the mixture standards of monosaccharide (**Fuc**, **Glc**, **Gal**, **GalA**) and oligosaccharides (laminaribiose, **DP2**; laminaritetraose, **DP4**); **S2**, the mixture standards of **Glc**, laminaribiose (**DP2**), laminaritriose (**DP3**), laminaritetraose (**DP4**), laminaripentaose (**DP5**), and laminarihexaose (**DP6**); **DEP**, the partial acid hydrolysates of dextran used as marker; **PGN, GGN, BGN, DEN**, and **STN**, enzymatic hydrolysates of polygalacturonic acid, guar galactomannan, oat glucan, dextran and starch with corresponding enzymes, respectively. The sample codes were the same as in Table S1.

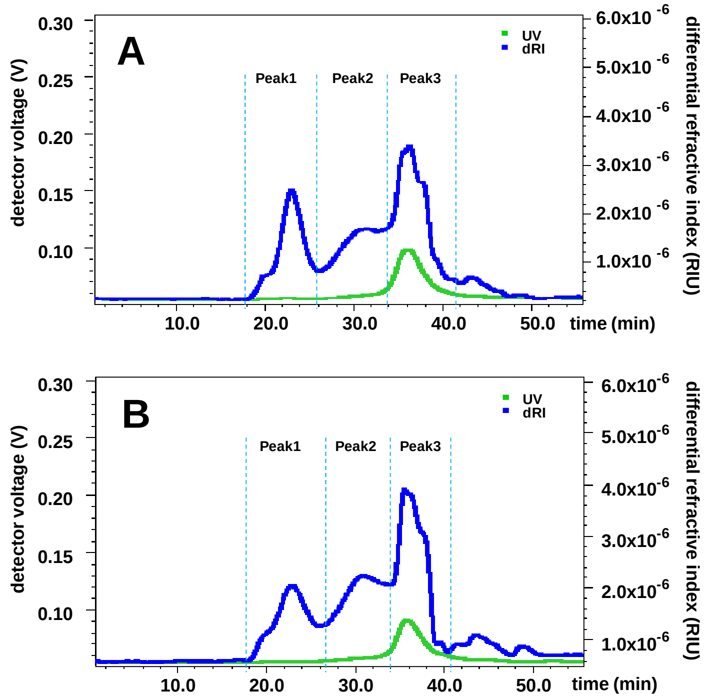

Fig. S5. Representative HPSEC chromatograms of polysaccharides from Bhutanese Cordyceps (A) and natural *C. sinensis* (B)

Table S1. The content of investigated nucleosides and their bases in Bhutanese Cordyceps and natural *Cordyceps sinensis*

Codes	Origins	Contents of investigated components (μg/g) [a]										
		Uracil	Cytidine	Guanine	Hypoxanthine	Adenine	Uridine	Thymine	Inosine	Guanosine	Thymidine	Adenosine
CS1	China	- (undetected)	-	256.1	308.5	366.6	2289.4	5.0	620.7	2044.9	48.8	2213.2
CS2	China	557.5	±	455.5	521.2	396.2	1684.2	65.4	488.7	1542.1	106.3	983.3
CS3	China	± (under LOQ)	±	526.1	18.0	607.1	4087.2	32.4	331.9	1822.0	136.2	2909.7
CS4	China	±	±	1007.4	58.5	1033.1	4521.0	219.8	478.6	2184.8	125.1	2847.7
CS5	China	±	±	486.5	±	571.7	3668.8	16.7	255.0	2042.8	99.8	2743.1
CS6	China	±	±	693.9	20.1	770.1	4216.1	68.8	314.6	1971.2	177.2	2818.5
CC1	Bhutan	±	±	264.3	275.9	489.7	4780.5	-	2915.7	3176.9	90.4	1352.1
CC2	Bhutan	99.5	±	412.6	400.6	952.3	6314.0	53.1	3455.8	3637.1	277.2	1781.7
CC3	Bhutan	223.0	-	546.0	729.8	789.8	7157.8	94.9	4203.4	3154.2	210.3	1225.2
CC4	Bhutan	97.2	±	90.7	112.5	163.9	2294.8	-	625.2	1062.0	-	980.9
CC5	Bhutan	±	±	379.9	245.6	515.0	4217.1	4.6	3113.3	3206.5	102.9	1128.5
CC6	Bhutan	43.8	±	663.4	59.0	1178.1	3573.3	80.0	532.3	1497.2	251.5	1849.9
CC7	Bhutan	400.2	228.5	2415.0	1525.7	2959.0	6446.2	530.8	5391.1	1144.8	107.7	941.2
CC8	Bhutan	94.7	42.2	895.0	120.6	1275.7	3938.4	210.8	634.8	1609.5	86.7	2115.3
CC9	Bhutan	53.1	34.6	765.1	82.3	1281.8	4417.6	135.6	588.2	2116.9	315.9	2486.1
CC10	Bhutan	41.4	±	623.8	165.5	842.7	4540.0	31.0	1099.6	2223.2	184.1	2249.0
CC11	Bhutan	195.3	55.4	1067.8	252.4	1323.2	5367.5	157.1	1825.5	2203.5	-	2763.3
CC12	Bhutan	48.1	-	121.4	284.8	304.2	2322.7	-	542.0	1199.9	-	1423.1

[a], all data were the average of two measurements with coefficient of variation < 3%.

Table S2. The molar ratios of compositional monosaccharides of polysaccharides from Bhutanese Cordyceps and natural *Cordyceps sinensis*

Sample Codes	Compositional monosaccharides and molar ratios [a]		
	Mannose	Glucose	Galactose
CS1	1.0	11.5	1.6
CS2	1.0	4.9	0.8
CS3	1.0	6.5	1.1
CS4	1.0	4.3	2.2
CS5	1.0	3.7	0.8
CS6	1.0	4.4	0.8
CC1	1.0	0.7	1.3
CC2	1.0	1.1	1.0
CC3	1.0	2.3	1.3
CC4	1.0	1.7	1.5
CC5	1.0	0.7	3.8
CC6	1.0	4.7	1.8
CC7	1.0	6.4	1.5
CC8	1.0	4.6	1.4
CC9	1.0	2.8	1.0
CC10	1.0	4.9	2.1
CC11	1.0	5.0	1.2
CC12	1.0	4.0	2.0

[a], all data were the average of two measurements with coefficient of variation < 5%;

The sample codes were the same as in Table S1;
